# Health-Related Quality of Life in Pediatric Patients with Leukemia in Singapore: A Cross-Sectional Pilot Study

**DOI:** 10.3390/ijerph16122069

**Published:** 2019-06-12

**Authors:** Carol C. Choo, Peter K.H. Chew, Pinhong Tan, Jessica Q. Choo, Amanda M.H. Choo, Roger C. Ho, Thuan Chong Quah

**Affiliations:** 1 Department of Psychology, College of Healthcare Sciences, James Cook University, Singapore 387380, Singapore; peter.chew@jcu.edu.au; 2 Department of Pediatrics, National University of Singapore, 1E Kent Ridge Road, Singapore 119228, Singapore; wnozer@gmail.com (P.T.); jessica-choo@hotmail.com (J.Q.C.); paeqtc@nus.edu.sg (T.C.Q.); 3 Cambridge Biomedical Campus, University of Cambridge School of Clinical Medicine, Cambridge CB2 0SP, UK; amhc2@cam.ac.uk; 4 Department of Psychological Medicine, Yong Loo Lin School of Medicine, National University of Singapore, 1E Kent Ridge Road, Singapore 119228, Singapore; pcmrhcm@nus.edu.sg; 5 Institute for Health Innovation and Technology (iHealthtech), National University of Singapore, Singapore 117599, Singapore; 6 Center of Excellence in Behavioral Medicine, Nguyen Tat Thanh University, Ho Chi Minh City 70000, Vietnam; 7 Faculty of Education, Huaibei Normal University, 100 Dongshan Road, Huaibei 235000, China

**Keywords:** Leukemia, pediatric, health-related quality of life, age

## Abstract

There has been a paradigm shift in health service delivery to a more holistic approach, which considers Quality of Life (QoL) and overall functioning. Health-Related Quality of Life (HRQoL) is a multidimensional construct that encompasses physical functioning as well as psychosocial aspects of emotional and social functioning. This study explored factors related to HRQoL in Asian pediatric patients with leukemia in Singapore. The available variables included: age, treatment duration, household income, gender, ethnicity, religion, diagnosis, and phase of treatment. It is hypothesized that the relationships will be significant. In the current study, there were 60 patients (60% males) with leukemia; their ages ranged from 1 to 21 years (Mean = 8.03, Standard Deviation = 4.55). The hypothesis was partially supported. Age had a significant positive relationship with physical functioning, *r*(60) = 0.28, *p* < 0.05, physical health, *r*(60) = 0.28, *p* < 0.05, and the total HRQoL score, *r*(60) = 0.29, *p* < 0.05. Treatment duration had a positive relationship with school functioning, *r*(60) = 0.28, *p* < 0.05. All other correlations were statistically non-significant. The effects of the available psychosocial variables of gender, ethnicity, and religion were examined on scores from the Pediatric Quality of Life Inventory (PedsQL). Ethnicity had a significant effect on social functioning, U = 292.00, *p* < 0.05, *r* = 0.3 (medium effect size). Specifically, Chinese (Median = 85.00, *n* = 33) had significantly higher scores on social functioning than others (Median = 70.00, *n* = 27). The remaining comparisons were statistically non-significant. The current findings added to QoL research, and provided an impetus for more research in the area of HRQoL for children with leukemia in Singapore.

## 1. Introduction

Health services are increasingly embracing holistic approaches to healthcare delivery, which consider Quality of Life (QoL) and overall functioning [1]. Due to this contemporary shift towards the recovery paradigm, QoL is becoming a crucial indicator of optimal healthcare. Health-Related Quality of Life (HRQoL) is the perception of the impact that illness and medical treatment have on one’s life [2]. HRQoL is a multidimensional construct, which encompasses physical functioning, as well as psychosocial aspects of emotional and social functioning [3]. HRQoL has been investigated in children, with a general consensus that children’s self-report of their own perceptions is optimal [3,4]. Reporting children’s own perception of HRQoL will assist parents, caregivers, health professionals, and policy makers to gain insight into their specific needs [5], which could inform holistic and targeted service delivery.

Review of relevant research suggested that children undergoing cancer treatment may experience reduced QoL, in the initial period after cancer diagnosis, prior to improving progressively, and again after the first year of treatment [6]. Deterioration of QoL in pediatric cancer patients was often attributed to painful clinical interventions, surgery, intensive chemotherapy, and hospitalization. Existing literature indicated that the physical, emotional, and social dimensions of the QoL of children with cancer differed according to various demographic factors, e.g., age [7] and gender [8], as well as disease and treatment-related factors, e.g., diagnosis, duration, and the type of treatment [9]. Specifically, children, as compared with adolescents, reported lower QoL for the initial period after diagnosis. In addition, recent literature showed that female children tended to report lower QoL than males, specifically in cognitive and emotional dimensions [10].

Conclusions concerning QoL drawn from prior studies conducted in the West are not easily generalizable to Asian populations due to the culturally sensitive nature of the construct of QOL [11]. Studies on HRQoL were done in children in the U.S. [3,4], Greece [10], and Australia [12] with various severe and chronic conditions. A number of recent studies done in Singapore underscored the influence of psychosocial variables on QoL [13,14,15]. Many of the psychosocial factors contributing to QoL were culture-specific. Local studies conducted in Singapore reiterated the importance of careful consideration of our unique sociocultural context [14,15,16], and cautioned against the assumption that conclusions drawn from research done in the West could be generalized to the local context [16]. In Asia, a recent exploratory cross-sectional study in Japan [17] evaluated the QoL of children with leukemia. It was found that QoL was significantly decreased for children with leukemia when compared with healthy controls, except in the area of emotional functioning. QoL differed with treatment phase, and it was concluded that QoL may change with the progression of leukemia treatment. A recent study done on a small sample of 32 local school-going children receiving treatment for cancer [18] found that the domains of HRQoL were affected differently, and this was attributed to the differences in treatment strategies and clinical course.

However, it remains unclear about the generalizability of the results to the local sample of children in Singapore with leukemia, regarding the specific contribution of demographic variables and treatment-related factors to HRQoL This research question is relevant and useful to healthcare clinicians working with the recovery paradigm in health service delivery, to assist in service planning for optimal holistic health outcomes in local children with leukemia. Pertinent research in the local population could inform greater capacity in clinicians to recognize demographic and treatment-related variables associated with deterioration of QoL, and to tailor early interventions accordingly, so as to minimize the impact on children’s QoL and their overall functioning.

Recent local research on adults suggested that various demographic factors, e.g., education level contributed to QoL in adult patients with chronic illness [13,15]. Recent large-scale local research also suggested that gender [19,20,21] and ethnicity [16] contributed to vulnerability towards poor mental health and overall wellbeing in Singaporeans, while religiosity was protective and contributed to resilience [16]. However, it is unclear if the finding is generalizable across the lifespan for children with leukemia. Other recent Asian studies suggested that treatment phase [17,18] contributed to HRQoL in children. Other recent relevant studies suggested that duration of illness [19] and treatment duration [10,15] may contribute to QoL [22], together with possible influences from demographic variables such as ethnicity [16], gender [10,19,20,21], and age [10,15,23]. However, the specific contribution of these demographic and treatment-related factors on HRQoL in children with leukemia in Singapore has not been fully explored.

This study aims to address the current gap in local literature by examining the available variables namely treatment-related variables and demographic characteristics such as ethnicity [16], age [10,15,24], gender [10,25], education [10,15,26], household income status [21], and religiosity [27], as well as treatment duration [13,15] and phase of treatment [17,18] in their relationships with HRQoL for pediatric patients with leukemia. The study will (1) explore bivariate relationships between the continuous variables, namely: age, treatment duration, monthly household income, and HRQoL in pediatric patients with leukemia (2) examine the effects of the available categorical variables, namely: gender, ethnicity, religion, diagnosis, and treatment phase on HRQoL. It is hypothesized that the relationships will be significant.

## 2. Methods

### 2.1. Procedure

Ethics approval was obtained from the Domains-Specific Review Board of a large teaching hospital in Singapore (IRB protocol#: DSRB 2013/01150). A sample of 60 pediatric patients diagnosed with leukemia were recruited at outpatient clinics, day therapy programs, and pediatric oncology wards at a large teaching hospital in Singapore from January to May 2010. Although the public hospital is geographically located in the west, the hospital receives referrals for delivery of a specialist multidisciplinary service for pediatric patients with leukemia from all regions in Singapore. Convenience sampling was employed for the cross-sectional pilot study. Eligibility criteria for the study included the following: (a) pediatric patients between 1 to 21 years of age diagnosed with leukemia (acute lymphoblastic leukemia, acute myeloid leukemia, or mixed-phenotype acute leukemia), (b) able to understand English and/or Mandarin, and (c) absence of co-morbidities. Exclusion criteria included (a) having other co-morbidities and (b) the absence of a parent/caregiver.

The patients’ subjective Health-Related Quality of Life (HRQoL) was investigated with the use of an adapted pictorial version of the Pediatric Quality of Life Inventory (PedsQL) 4.0 [4]. Demographic information collected included: age, gender, ethnicity, religion, diagnosis, treatment phase and duration, monthly household income, and primary caregiver’s education status.

Clinical staff conducted the recruitment. After parental consent and participants’ assent was obtained, the questionnaires were then administered via an iPad. After completion of the questionnaires, participants and parents/caregivers were thanked for their participation.

In the current study, there were 60 patients (60% males) diagnosed with leukemia. Their ages ranged from 1 to 21 years (Mean (M) = 8.03, Standard Deviation (SD) = 4.55). The demographics and characteristics of the participants are presented in Table 1.

### 2.2. Materials

The survey included items on demographic background and the following questionnaire. The questionnaire was adapted into a pictorial format, with reference to PedsQL 4.0 [4], by a team of clinicians and an illustrator who had previous experience with pictorial books for pediatric oncology patients. Considerations were made to ensure the questionnaire was understandable and relatable to patients of different nationalities, ethnicities, ages, and stage of treatment. Cultural bias was minimized in all aspects including symbols, activities, and food depicted. Pictorial representations of facial expressions and body language were depicted in a universally relatable manner. To ensure that patients understood how to answer the questionnaire, a sample question was included.

The PedsQL 4.0 [4] is one of the most commonly used measure for exploring Health-Related Quality of Life, HRQoL in children [28], with well-established validity and reliability [3,4,29]. It has been used to investigate HRQoL for local school-going children undergoing cancer treatment [18]. It comprises multiple age versions of child self-report and proxy (parent/caregiver) report, that vary only on the language used in each form, ensuring developmental appropriateness [4,30]. The PedsQL consists of scales measuring various dimensions of functioning (physical, emotional, social, and school) and individuals respond with the extent to which each item has been an issue for them within the last month. To ensure the scale was age appropriate, children aged 8 years and above responded to five-point Likert scales, while children aged 7 years and below respond to three-point Likert scales, incorporating pictures to assist them. Scores are transformed to a 0–100 scale. The transformed scores are averaged for each scale or a set of scales, to provide the following summary scores: Psychosocial Health Summary Score (the mean of emotional, social and school functioning scales), Physical Health Summary (physical functioning scale), School Health Summary (school functioning scale), and Total Summary Score (the mean of all scales). Higher scores indicate better HRQOL.

## 3. Results

The data were analyzed using SPSS Version 25 (IBM Corp., Armonk, NY, USA) with the alpha level set at 0.05. The description of the HRQoL scores on the Pediatric Quality of Life Inventory (PedsQL) for the sample of 60 children is presented in Table 2.

A series of Pearson product-moment correlation coefficient was conducted to explore the bivariate relationships between age, treatment duration, monthly household income, and scores from the PedsQL. Age had a significant positive relationship with Physical Functioning, *r*(60) = 0.28, *p* < 0.05, Physical Health, *r*(60) = 0.28, *p* < 0.05, and the Total Scale Score on the PedsQL, *r*(60) = 0.29, *p* < 0.05. Treatment duration had a positive relationship with School Functioning, *r*(60) = 0.28, *p* < 0.05. All other correlations were statistically non-significant. 

A series of three Mann-Whitney U Tests were conducted to examine the effects of the available psychosocial variables of Gender (Males versus Females), Ethnicity (Chinese versus Others), and Religion (Yes versus No) on scores from the PedsQL. Ethnicity had a significant effect on Social Functioning, U = 292.00, *p* < 0.05, *r* = 0.3 (medium effect size). Specifically, Chinese (Median = 85.00, *n* = 33) had significantly higher scores on Social Functioning than Others (Median = 70.00, *n* = 27). The remaining comparisons were statistically non-significant. A series of two Kruskal-Wallis Tests was conducted to examine the effects of Diagnosis (B-Cell ALL versus T-Cell ALL versus AML/MPAL) and Treatment Phase (Maintenance versus Completed Treatment versus Induction versus Others) on PedsQL scores. All comparisons were statistically non-significant. The Mann-Whitney U Tests and the Kruskal-Wallis Tests are presented in Table 3.

## 4. Discussion

This study explored factors related to HRQoL in Asian pediatric patients with leukemia in Singapore. The available demographic and treatment-related variables included: age, treatment duration, household income, gender, ethnicity, religion, diagnosis, and phase of treatment. It is hypothesized that the relationships would be significant. The hypothesis was partially supported. Age had a significant positive relationship with physical functioning, physical health, and the total HRQoL score. Treatment duration had a positive relationship with school functioning. Ethnicity had a significant effect on social functioning; specifically, Chinese had significantly higher scores on social functioning than others. The comparisons were statistically non-significant for all other variables. However, the results should be interpreted with caution. Given the exploratory nature of the study, the Type I error was not controlled for using the Bonferroni adjustment. Once controlled, the results could be non-significant.

The finding that the effects of diagnosis and treatment phase on HRQoL scores were not significant was contrary to another study done in Japan [17] for children with leukemia, suggesting that HRQoL may vary across different Asian nationalities, further re-iterating the importance of local research in this important health topic. The findings of the non-significant results are not consistent with previous studies done in Singapore on adult patients with chronic mental illness [13], indicating that QoL may vary across the lifespan and may differ between physical and mental illness. However, the non-significant findings are consistent with a recent study [10] which found that QoL of Greek children did not change during leukemia treatment.

The results show that age had a significant positive relationship with physical functioning and physical health, and the total HRQoL score was consistent with a recent study [10] that found that factors prominent in HRQoL of Greek children with leukemia included older age. However, it is unclear what contributed to better QoL in older children, e.g., increased capacity to manage pain [10], or the development of resilience and coping for different stressors across the lifespan [25]. The finding offers an impetus for further investigations into HRQoL in younger children with leukemia. Future research could employ a qualitative approach and interview younger children as well as their parents/siblings and clinical staff for corroboratory information on the factors affecting HRQoL, and the factors contributing to vulnerabilities and resilience in disease management.

In view of the importance of educational attainment and academic performance in Asian contexts [25], there have been concerns that school-going children undergoing cancer treatment may experience long absences from school, thus affecting their school functioning [18]. The finding that treatment duration had a positive relationship with school functioning provided preliminary results to support the importance of treatment compliance for the entire duration of the chronic illness. The findings are consistent with the review of relevant research that suggested that children undergoing cancer treatment may experience reduced QoL during the initial 3 to 6 months after cancer diagnosis, prior to improving progressively, about 6 months after diagnosis, and again after completion of the first year of treatment [6].

The finding that ethnicity had a significant effect on social functioning is consistent with local large-scale research underscoring the strengths and vulnerabilities of Singapore’s diverse ethnic cultures [16]. The finding offers an impetus for further investigations into social functioning for children from non-Chinese ethnicities. Future research could employ a qualitative approach and interview children from a range of different ethnicities, as well as their parents/siblings and peers for corroboratory information about the factors contributing to vulnerabilities and resilience in social functioning for children with leukemia.

## 5. Limitations

Some limitations of the present study need to be considered. These include the lack of an adequate control condition, preventing any comparisons from being done. Hence, future studies should aim to match clinical data with data collected from healthy populations to provide a more holistic interpretation of potential variables influencing QoL. Given the cross-sectional design of the study, causality was unable to be demonstrated; future research should consider a longitudinal design to understand how QoL changes in relation to various variables over time. Furthermore, the questionnaire used in the pilot study was a pictorial version adapted with reference to PedsQL 4.0 [4]. As the study was a pilot study with a small sample size of 60, the sample size was too small for validation to be conducted on the adapted instrument. Due to the small sample size, it is also not statistically feasible to examine how the different types of leukemia might impact QoL. The research was conducted at one local hospital, which also limited the generalizability of the results. Future research could endeavor to sample patients from other private and public hospitals in Singapore, and to employ stratified sampling strategy, as well as increase the sample size, to be more representative of the local population for a validation study. The validation of the adapted instrument to measure HRQoL, as well as the contribution of leukemia types to HRQoL could be investigated in future studies with a larger sample of pediatric patients with leukemia.

In addition, a review of relevant research suggested that children undergoing cancer treatment might experience reduced autonomy, poor psychological status, and depression, especially during the initial 3 to 6 months after cancer diagnosis which could result in reduced HRQoL [10]. A wide range of psychosocial factors might impact on patients, previously investigated but not included in the current study, e.g., self-efficacy, coping, family and social support [13,26,31], and self-esteem [32]. Other psychosocial variables that clinical psychologists often consider when working with children and their families may include systemic factors that may predispose and increase the child’s vulnerability [33]. Such factors may include family history of mental illness and early patterns of attachment and bonding. The aforementioned variables could be extraneous variables. In view of the proliferation of recent research underscoring the importance of psychosocial interventions for both physical and mental illness across the lifespan [14,25,34], such information will be important as they would have implications for the role that allied health professionals play in the delivery of psychosocial interventions targeted towards enhancing resilience, coping, and self-efficacy in children with leukemia. While such information was not collected in this current study, future research could employ in-depth interviews to elicit the intricate interplay among psychosocial factors and HRQoL, which could be confounding variables in this study. In view of the prevalence in co-morbidity between physical and mental illness [14], there is indication that co-morbid depression could be investigated in future research, e.g., with the use of an appropriate tool such as the Children Depression Inventory [35] to assess for depressive symptoms.

## 6. Conclusions

This pilot study explored factors related to HRQoL in Asian pediatric patients with leukemia in Singapore. Results indicated that age had a significant positive relationship with physical functioning, physical health, and the overall HRQoL of pediatric patients with leukemia. Treatment duration had a positive relationship with school functioning. Ethnicity had a significant effect on social functioning; specifically, Chinese had significantly higher scores on social functioning than others. The comparisons were statistically non-significant for all other variables. The current findings were interpreted in the context of other studies in the area. The outcomes from the pilot study added to QoL research, and provided an impetus for more research in the area of HRQoL, with local pediatric patients with severe and chronic disorders, namely, leukemia.

## Figures and Tables

**Table 1:** Characteristics of the Sample.

Variables	M	SD
Age	8.03	4.55
Patient treatment duration (in months)	27.77	31.01
Monthly household income (SGD)	2137.90	1913.49
	*n*	%
Gender		
Male	36	60.0
Female	24	40.0
Ethnicity		
Chinese	33	55.0
Others (Malay, Indian, etc.)	27	45.0
Religion		
Yes	54	90.0
No	6	10.0
Diagnosis		
B-Cell ALL	40	66.7
T-Cell ALL	11	18.3
AML/MPAL	9	15.0
Treatment Phase		
Maintenance	20	33.3
Completed Treatment	15	25.0
Induction	13	21.7
Others (Palliative, Post-Transplant, etc.)	12	20.0

ALL = acute lymphoblastic leukemia; AML = acute myeloid leukemia; MPAL = mixed-phenotype acute leukemia. M = Mean; SD = Standard Deviation.

**Table 2:** Description of the Scores on Pediatric Quality of Life Inventory (PedsQL).

Variables	1	2	3	4	5	6	7
Scales							
1. Physical Functioning	-						
2. Emotional Functioning	0.75 ***	-					
3. Social Functioning	0.36 **	0.40 **	-				
4. School Functioning	0.60 ***	0.57 ***	0.42 **	-			
Summary Scores							
5. Physical Health	1 ***	0.75 ***	0.36 **	0.60 ***	-		
6. Psychosocial Health	0.75 ***	0.86 ***	0.69 ***	0.82 ***	0.75 ***	-	
7. Total Scale	0.92 ***	0.87 ***	0.56 ***	0.76 ***	0.92 ***	0.94 ***	-
							
M	70.42	64.42	74.71	52.64	70.42	64.51	66.74
SD	23.26	21.79	20.50	25.19	23.26	17.74	18.37

** *p* < 0.01, *** *p* < 0.001.

**Table 3:** Mann-Whitney U Tests for Gender, Ethnicity, and Religion, and Kruskal-Wallis Tests for Diagnosis and Treatment Phase on Pediatric Quality of Life Inventory (PedsQL) Scores.

Scales	Mann-Whitney U			Kruskal-Wallis H	
	Gender	Ethnicity	Religion	Diagnosis	Treatment Phase
Physical Functioning	381.00	440.50	153.50	5.46	1.36
Emotional Functioning	414.00	362.50	143.50	4.25	0.64
Social Functioning	367.00	292.00 *	103.50	3.65	3.78
School Functioning	356.50	361.00	143.50	1.06	1.06
Physical Health	381.00	440.50	153.50	5.46	1.36
Psychosocial Health	379.50	330.00	129.00	3.30	2.77
Total Scale	366.00	381.00	136.50	4.27	2.53

* *p* < 0.05.
